# Idiopathic normal pressure hydrocephalus and frontotemporal dementia: an unexpected association

**DOI:** 10.1093/braincomms/fcac319

**Published:** 2022-12-14

**Authors:** Adrien de Guilhem de Lataillade, Claire Boutoleau-Bretonnière, Jesus Aguilar-Garcia, Amandine Pallardy, Edith Bigot-Corbel, Vincent Roualdes, Julie Leroy, Philippe Damier, Hélène Pouclet-Courtemanche

**Affiliations:** Centre d'investigations cliniques, INSERM 1413, CHU Nantes, 44093 Nantes, France; Department of Neurology, CHU Nantes, 44093 Nantes, France; Nantes University, 44093 Nantes, France; Centre d'investigations cliniques, INSERM 1413, CHU Nantes, 44093 Nantes, France; Department of Memory Resource and Research Centre, CHU Nantes, 44093 Nantes, France; Department of Neurology, CHU Nantes, 44093 Nantes, France; Department of Neuroradiology, CHU Nantes, 44093 Nantes, France; Department of Nuclear Medicine, CHU Nantes, 44093 Nantes, France; Laboratory of Biochemistry, CHU Nantes, 44093 Nantes, France; Nantes University, 44093 Nantes, France; Department of Neurosurgery, CHU Nantes, 44093 Nantes, France; Department of Neurology, CHU Nantes, 44093 Nantes, France; Centre d'investigations cliniques, INSERM 1413, CHU Nantes, 44093 Nantes, France; Department of Neurology, CHU Nantes, 44093 Nantes, France; Nantes University, 44093 Nantes, France; Centre d'investigations cliniques, INSERM 1413, CHU Nantes, 44093 Nantes, France; Department of Memory Resource and Research Centre, CHU Nantes, 44093 Nantes, France; Department of Neurology, CHU Nantes, 44093 Nantes, France

**Keywords:** behavioural variant of frontotemporal lobar degeneration, Alzheimer’s disease, idiopathic normal pressure hydrocephalus

## Abstract

Idiopathic normal pressure hydrocephalus has a complex multifactorial pathogenesis and is associated with Alzheimer’s disease in many patients. To date, it is not well known if a similar association exists with behavioural variant of frontotemporal lobar degeneration. In a first step, we compare the prevalence of idiopathic normal pressure hydrocephalus in two groups of patients, one with behavioural variant of frontotemporal lobar degeneration (*n* = 69) and the other with Alzheimer’s disease (*n* = 178). In the second step, we describe more precisely the phenotype of patients with the association of idiopathic normal pressure hydrocephalus and behavioural variant of frontotemporal lobar degeneration. Firstly, we report that the prevalence of idiopathic normal pressure hydrocephalus was far higher in the group of patients with behavioural variant of frontotemporal lobar degeneration than in the group of patients with Alzheimer’s disease (7.25% and 1.1%, respectively, *P* = 0.02). Secondly, we show that patients with the double diagnosis share common clinical and para-clinical features of both idiopathic normal pressure hydrocephalus and behavioural variant of frontotemporal lobar degeneration patients, including CSF shunting efficacy in real-life experience. Overall, our results suggest a link between these two conditions and should encourage neurologists to look for idiopathic normal pressure hydrocephalus in their behavioural variant of frontotemporal lobar degeneration patients in the event of gait disturbances; the benefit/risk balance could indeed be in favour of shunt surgery for selected patients with this newly described entity.

## Teaser

De Guilhem de Lataillade *et al.* report an unexpected higher prevalence of idiopathic normal pressure hydrocephalus in a group of patients with behavioural variant of frontotemporal lobar degeneration than in a group with Alzheimer’s disease. The results suggest a possible pathophysiological link between these entities initially thought to be unrelated.

## Graphical Abstract

**Graphical abstract:**
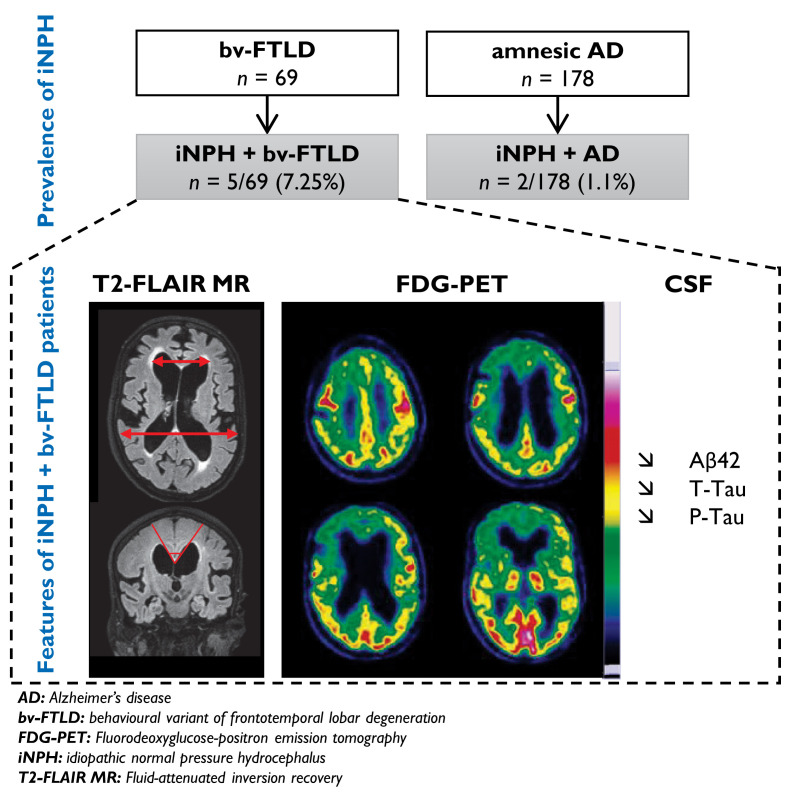

## Introduction

Idiopathic normal pressure hydrocephalus (iNPH) is a progressive neurological syndrome characterized by gait impairment, cognitive disturbances and urinary symptoms, associated with a disproportionately enlarged subarachnoid space hydrocephalus (DESH).^1,2^ This is a frequent condition since the prevalence could exceed 3% beyond 65 years old and is near 10% in the elderly.^3^ Invasive treatment, i.e. lumboperitoneal, ventriculoatrial (VAS) or ventriculoperitoneal shunt (VPS), is indicated after a complete assessment of the benefit/risk balance.

The idiopathic origin of iNPH is debated. Some authors have recently postulated that most iNPH cases are actually a consequence of a neurodegenerative process,^4^ mainly Alzheimer’s disease.^5,6^ The frequent deterioration of cognition observed during the follow-up of iNPH patients, even after shunt surgery,^6,7^ supports such a view. To our knowledge, the association with the behavioural variant of frontotemporal lobar degeneration (bv-FTLD) has previously been reported in only one case report.^8^

In the present study, we investigated the prevalence of iNPH in our yearly database of Alzheimer’s disease and bv-FTLD patients. In the second step, we described the clinical, morphological and functional imaging and biological features of iNPH associated with bv-FTLD.

## Materials and methods

### Assessment of iNPH prevalence

#### Patients

Patients with probable Alzheimer’s disease with amnestic presentation defined following the 2011 National Institute on Aging and Alzheimer's Association (NIA-AA) criteria^9^ and patients with possible or probable bv-FTLD defined by the 2011 Rascovsky’s criteria,^10^ seen between 1 January and 31 December 2019 in the MRRCN (Memory Resource and Research Centre of Nantes), were included. Patients with a history of head trauma with parenchymal consequences, clinical stroke, meningitis or subarachnoid haemorrhage, ≥4 lacunar infarcts, cerebral amyloid angiopathy, alcoholism, an association with Lewy body disease or Parkinson disease were excluded. All patients underwent a standardized evaluation including clinical examination, full neuropsychological assessment by a neuropsychologist and brain imaging (MRI or CT scan). When needed for diagnosis, patients had functional imaging [18-F-fluorodeoxyglucose PET (FDG-PET) or hexa-methyl-propyl-amineoxime-single photon emission computed tomography (HMPAO-SPECT)] and lumbar puncture (LP). Data were harvested from the care software of MRRCN. All data were collected with the informed consent of patients under institutional board approval.

#### Idiopathic normal pressure hydrocephalus diagnosis

Clinical symptoms and brain imaging data were analysed by two neurologists (Adrien de Guilhem de Lataillade and Hélène Pouclet-Courtemanche). The diagnosis was based on the American-European guidelines (except for the criterion of CSF opening pressure).^11^ We required the gait or balance impairment to have a marked impact on daily life.

### Investigation of idiopathic normal pressure hydrocephalus associated with frontotemporal dementia

#### Patients

We included all the patients with the association of iNPH and bv-FTLD (iNPH-bv-FTLD patients) seen in 2019 and 2020 at the MRRCN. In addition, we included an equal number of consecutive patients with bv-FTLD (with no criteria for iNPH) and iNPH patients (with the same diagnostic criteria as above and no criteria for Alzheimer’s disease or bv-FTLD) seen during this same 2019–20 period to obtain comparable samples.

#### Clinical and neuropsychological assessments

All selected patients had a complete thorough examination, including clinical, neurological and neuropsychological evaluations. When indicated (probable iNPH diagnosis, no contra-indication for LP or shunting), a tap test with three subtractive LPs (40 ml) were performed on three successive days. In order to evaluate clinical improvement, patients were assessed, before and the day after the third LP, for walking by a physiotherapist [the improvement was measured with the CGI-C (Clinical Global Impression of Change), in which scores range from 1 (very much improved) to 4 (no change), from a video-recorded gait examination (assessment of stride length and height, balance, swing, walking cadence and stride numbers) on a 10-m round-trip] and for cognition [MMSE (mini-mental state examination), MoCA (Montreal Cognitive Assessment) and FAB (Frontal Assessment Battery)]. In the case of a shunt, the CGI-C for walking was assessed 1–3 months after the surgery.

#### Imaging analyses

All patients had MRI, except for three iNPH patients who had CT scan. In the iNPH-bv-FTLD and bv-FTLD groups, most patients also had FDG-PET or HMPAO-SPECT. An analysis, blinded to clinical features, of morphological and functional imaging was made by a radiologist (Jesus Aguilar-Garcia) and a nuclear physician (Amandine Pallardy), respectively. For functional imaging, a visual/qualitative analysis was firstly done combining HMPAO-SPECT and FDG-PET data, then a quantitative comparison of the mean activity of the volume of interest (VOI) was made from FDG-PET imaging only; all these analyses were realized after cerebellar normalization.

### Cerebrospinal fluid analysis

Cerebrospinal fluid was collected according to our standardized procedure (see Supplemental Data). CSF Aβ_40_, Aβ_42_, total Tau (T-tau) and phosphorylated Tau^181P^ (P-Tau) were measured using a commercially available sandwich enzyme-linked immunosorbent assay (INNOTEST; Fujirebio Europe NV, Gent, Belgium) according to the manufacturer’s instructions. To compare measures made at different periods, the CSF biomarker levels were expressed relative to the cut-offs applied at the moment of the collection.

### Statistical analysis

Qualitative variables were expressed as frequencies and percentages. Quantitative variables were expressed as median, interquartile range (IQR25-75) or mean, standard deviation. In the first part of the study, Fisher’s exact test was used for qualitative variables; Student’s and Mann–Whitney test were used for quantitative variables. In the second part of the study, Fisher’s exact test was used for comparing qualitative variables; Kruskal–Wallis’ test for ordinal qualitative variables; and Mann–Whitney test for quantitative variables. Outliers were excluded if identified by Grubb’s test. Statistical testing was done at the two-tailed α level of 0.05. Data were analysed using BiostaTGV (https://biostatgv.sentiweb.fr/) and GraphPad Prism.

## Results

### Prevalence of iNPH is higher among bv-FTLD than Alzheimer’s disease patients

**Figure 1:**
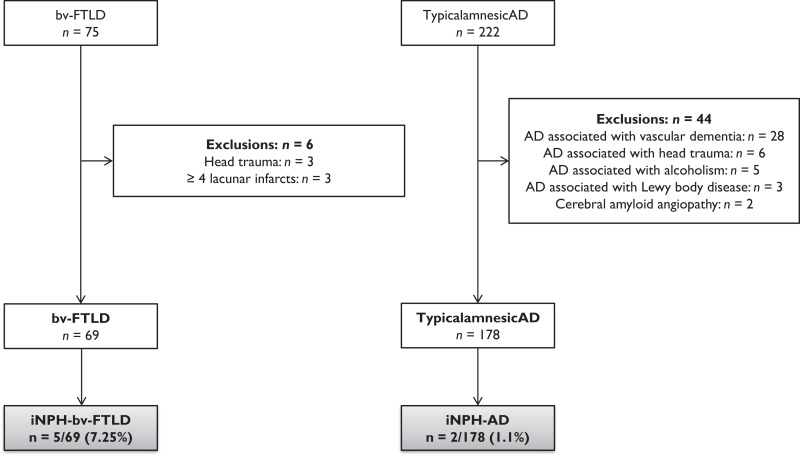
**Flow chart**. iNPH was diagnosed in 5 (7.25%) of the 69 bv-FTLD patients and in 2 (1.1%) of the 178 Alzheimer’s disease patients seen in 2019 (*P* = 0.02, Fisher’s exact test)

In 2019, 75 patients with bv-FTLD and 222 with Alzheimer’s disease were seen in MRRCN. After the application of exclusion criteria, 69 patients with bv-FTLD and 178 with Alzheimer’s disease were compared for iNPH prevalence (Fig. 1). The diagnosis of bv-FTLD was quoted as possible for 11 (15.9%), probable for 54 (78.3%) and genetically confirmed for four patients (5.8%; three with a *C9Orf72* expansion, and one with a *GRN* mutation). Two bv-FTLD patients (2.9%) had amyotrophic lateral sclerosis (ALS). Alzheimer’s disease patients were significantly older (73.1 ± 8.3) than bv-FTLD patients (65.7 ± 8.3) (*P* < 0.0001). MMSE at diagnosis was similar in the two groups (23.0 ± 4.3 and 21.9 ± 5.3) (Supplementary Table 1). Clinical and radiological criteria for iNPH were met in five (7.25%) bv-FTLD patients and two (1.1%) Alzheimer’s disease patients (*P* = 0.02) (Fig. 1). No patient among the 44 excluded Alzheimer’s disease subjects met the iNPH diagnostic criteria.

### Clinical, imaging and biological features of iNPH associated with bv-FTLD

Nine patients meeting the diagnostic criteria for both iNPH and bv-FTLD were seen in 2019 (five) and 2020 (four) at the MRRCN and were compared to nine bv-FTLD and nine iNPH patients (Supplementary Table 2).

### iNPH-bv-FTLD patients compared to iNPH and bv-FTLD patients

iNPH-bv-FTLD patients differed from iNPH patients solely in terms of their cognitive profile. In the iNPH group, some patients met one or two of Raskovsky’s clinical criteria but none had perseverations and only one had hyperorality (Supplementary Table 2).

**Figure 2:**
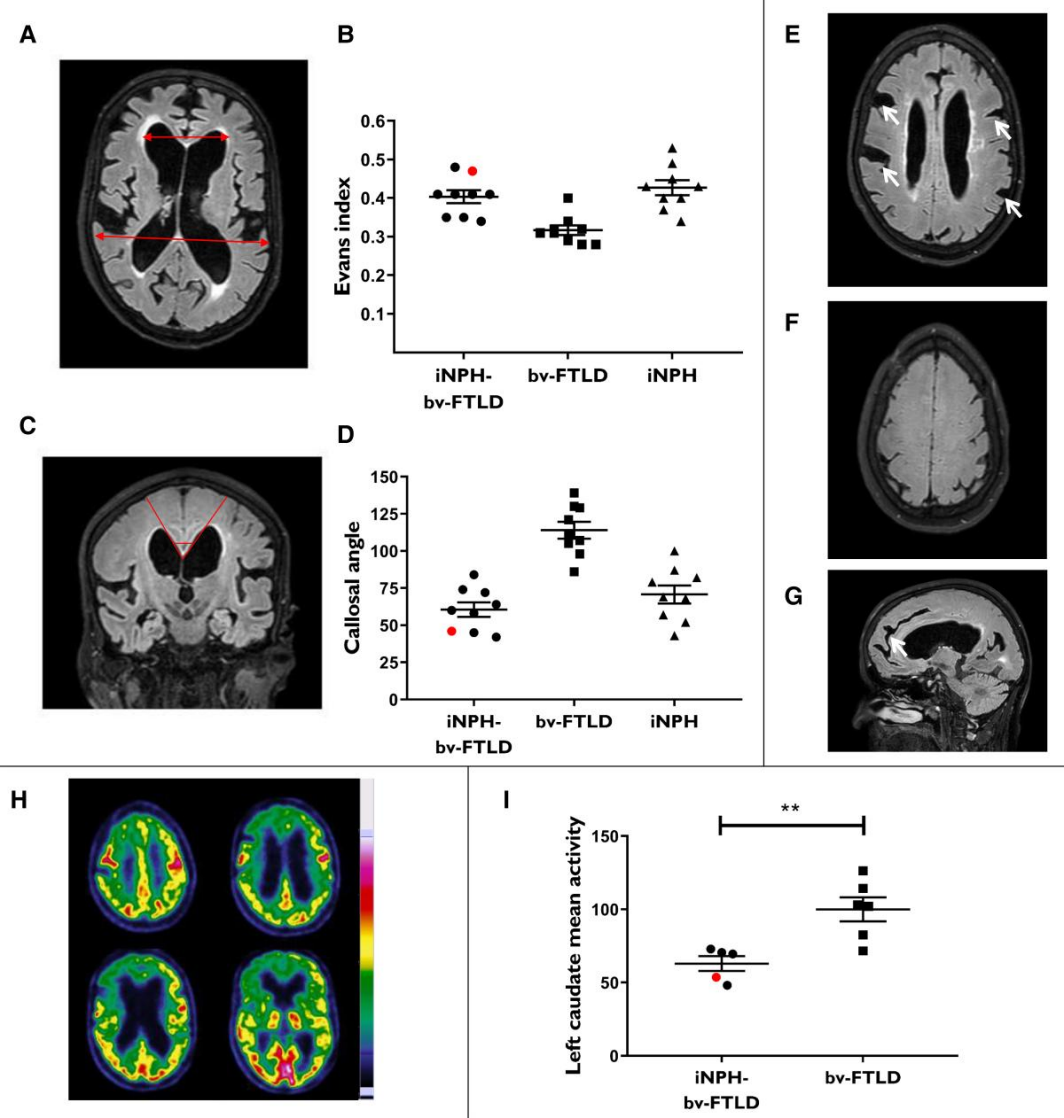
**Imaging features of iNPH-bv-FTLD patients**. (**A**) Axial T2-FLAIR MR image of an iNPH-bv-FTLD patient, showing frontotemporal asymmetrical atrophy, with a right predominance and ventriculomegaly with two segments (red arrows) used to calculate the Evans index. (**B**) Evans index for all patients of the three groups. (**C**) Coronal T2-FLAIR MR image of the same patient, showing a sharp callosal angle (red lines) and a convexity of the third ventricular walls. (**D**) Callosal angle (degrees) for all patients of the three groups. (**E–G)** Other T2-FLAIR MR images of the same iNPH-bv-FTLD patient: focal dilated sulci ((**E**), arrows), tight superior sulci at the convexity (**F**), cingulate sulcus sign ((**G**), arrow). (**H**) Example of axial slices of 18F-FDG PET scans in one patient, displayed with a rainbow colour scale, after metabolic normalization to the putamen. They show a diffuse hypometabolism of the subcortical and striatal structures that might be secondary to the iNPH, although the anterior and right predominance is consistent with an association with bv-FTLD. (**I**) As an example of basal ganglia hypometabolism, quantification of mean left caudate activity is shown for patients who had FDG-PET imaging. The values are normalized to the mean value of the bv-FTLD group. **P < 0.01 (Mann–Whitney test, *U* = 1). On each graph, the red symbol corresponds to the quantitative value of the illustrative patient

iNPH-bv-FTLD patients differed from bv-FTLD patients in terms of MRI features (Fig. 2A–G, Supplementary Table 3). In the latter group, only dilated Sylvian fissures and enlargement of the temporal horns were observed. Gait or balance impairment was not observed among bv-FTLD patients, and sphincter disorder was observed in only one bv-FTLD patient (Supplementary Table 2).

Frontotemporal atrophy or hypometabolism was observed in most iNPH-bv-FTLD and bv-FTLD patients, but were also found in some iNPH patients (Fig. 2A, C and H, Supplementary Tables 3 and 4).

### Deep brain region hypometabolism in iNPH-bv-FTLD patients

Combining HMPAO-SPECT and FDG-PET qualitative analyses, severe periventricular hypoperfusion/metabolism was observed more frequently in the iNPH-bv-FTLD group than in the bv-FTLD group (75% and 11.1%, respectively, *P* < 0.01); basal ganglia hypoperfusion/metabolism was observed in all iNPH-bv-FTLD patients but in only half (55.5%) of the bv-FTLD patients (ns, *P* = 0.08) (Supplementary Table 4). Data from iNPH patients were too sparse (only four patients) to allow statistical comparison, but 50% and 75% of them showed severe periventricular and basal ganglia hypoperfusion/metabolism, respectively. Quantitative analyses of FDG-PET imaging data showed that basal ganglia mean activity was indeed significantly lower in the iNPH-bv-FTLD group compared to the bv-FTLD group, (*P* < 0.05 for all basal ganglia VOIs; for right caudate and putamen, *P* = 0.052) (Fig. 2H and I, Supplementary Fig. 1).

### CSF abnormalities in iNPH-bv-FTLD patients

**Figure 3:**
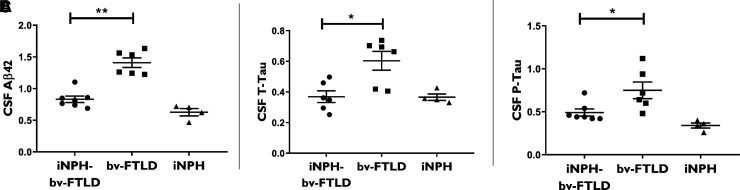
**CSF Aβ_42_ (A), T-Tau (B) and P-Tau (C) in iNPH-bv-FTLD (*n* = 7/9), bv-FTLD (*n* = 6/9) and iNPH (*n* = 4/9) groups**. To compare measures made at different periods, the CSF biomarker levels were expressed relative to the cut-offs applied at the moment of the collection. One T-Tau value from the iNPH-bv-FTLD group was excluded as it was identified as an outlier. iNPH-bv-DLFT versus bv-DLFT (Mann–Whitney test): (**A**) ** *P* < 0.01 (*U* = 0); (**B**) *P < 0.05 (*U* = 4); (**C**) *P < 0.05 (*U* = 4.5)

CSF Aβ_42_, T-Tau and P-Tau were significantly lower in the iNPH-bv-FTLD group than in the bv-FTLD group (*P* < 0.01, < 0.05 and <0.05, respectively) (Fig. 3). No patient had an Alzheimer’s disease CSF biomarkers profile.

### Tap test and CSF derivation improved gate disturbances in iNPH-bv-FTLD patients

**Figure 4:**
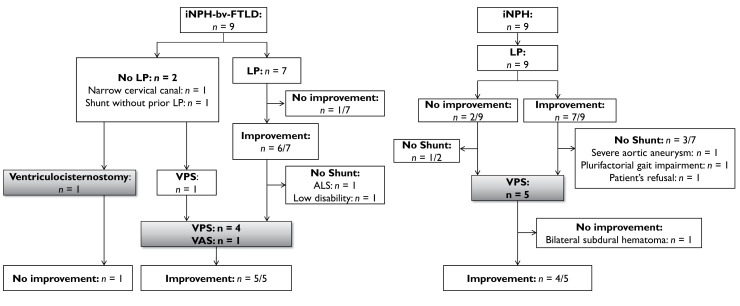
**Flow-chart presenting the effect of tap test (subtractive lumbar punctures) and shunt on gait in patients in the iNPH-bv-FTLD and iNPH groups**. The effect on gait was assessed 3 months after shunt surgery. LP, lumbar puncture; ALS, amyotrophic lateral sclerosis; VPS, ventriculoperitoneal shunt; VAS, ventriculoatrial shunt

In the iNPH-bv-FTLD and iNPH groups, tap test led to an improvement of gait disturbance: in six out of seven patients (85.7%) and seven out of nine patients (77.8%), respectively (Fig. 4). The level of improvement measured with the CGI-C was similar in both groups (Supplementary Table 2).

Five iNPH-bv-FTLD patients had shunt surgery (four VPS and one VAS): walking had improved in all patients at the 3-month post-surgery evaluation (median CGI-C = 2, range 1–4). One patient had a ventriculocisternostomy without any improvement (Fig. 4). Median follow-up of the five patients was 16 months; one had functional worsening, mainly because of cognitive decline 22 months after surgery, the other four patients were still benefitting from their shunt surgery at 3-, 5-, 16- and 24-month follow-up examinations (Supplementary Fig. 2).

## Discussion

Here, we report for the first time a high prevalence of iNPH cases among a bv-FTLD population, a prevalence far higher than that observed in a group of Alzheimer’s disease patients followed in the same Centre. Reinforcing our observation, the mean age (age being the main risk factor for iNPH)^3^ was higher in the Alzheimer’s disease group than in the bv-FTLD group.

iNPH-bv-FTLD patients share classical features of both iNPH patients (i.e. Adam and Hakim’s triad, hydrocephaly with DESH criteria on MRI,^12^ cingulate sulcus sign,^13^ walking improvement after CSF derivation) and bv-FTLD patients (i.e. behavioural disturbances, frontal and/or temporal atrophy, non-Alzheimer’s disease CSF biomarkers and for one patient ALS). Interestingly, an improvement of gait (but not of behavioural, cognitive and/or sphincter disturbances) was observed among the five iNPH-bv-FTLD patients 3 months after shunt surgery, an improvement that was maintained over time for some of them. The absence of improvement after ventriculocisternostomy in one patient does not rule out the diagnosis of iNPH associated with bv-FTLD, given that this procedure is known to be less effective than VPS or VAS.^14^ These observations should encourage clinicians to consider shunting in order to improve gait in iNPH-bv-FTLD patients. Moreover, shunting in the event of a comorbid neurodegenerative condition is currently debated in the field of Alzheimer’s disease;^15^ there are conflicting results from prospective studies trying to correlate gait and cognitive short-term outcomes after CSF depletion, with either biopsy samples^16,17^ or amyloid-PET imaging assessment of the amyloid burden.^18,19^ In the current state of knowledge, it seems reasonable to consider the improvement that CSF shunting could provide, at least on gait symptoms.

It is surprising that an association between bv-FTLD and iNPH has not previously been reported. The observation of ventriculomegaly, attributed to subcortical atrophy in bv-FTLD, might have been neglected. The systematic analysis of iNPH criteria in our bv-FTLD cohort probably allowed us to evidence this previously unknown association. Interestingly, a recent study reported a 1.6% prevalence of *C9orf72* expansion (one major genetic cause of bv-FTLD) in an iNPH group, whereas no such expansion was observed in the control group,^20^ suggesting a genetic link between iNPH and bv-FTLD.

Our study has several limitations. First of all, the diagnoses of iNPH and bv-FTLD were based on clinical and radiological criteria, despite iNPH criteria still being a subject of discussion,^21^ and bv-FTLD was not ascertained on pathological data. The risk here is to confound the coincidence of the two diseases with either a ‘behavioural variant of iNPH’ or an asymptomatic atrophy-linked ventriculomegaly in bv-FTLD patients. The first hypothesis is not supported by the observation in a majority of iNPH-bv-FTLD patients, but not among our iNPH patients group, of hyperorality and perseverations, which are quite specific behavioural symptoms of bv-FTLD.^22^ Furthermore, these symptoms are unusual among neuropsychiatric manifestations observed in isolated iNPH patients (i.e. apathy, sleepiness, depression and anxiety).^23–25^ Concerning the atrophy-linked ventriculomegaly hypothesis, DESH signs on morphological imaging are not seen in isolated bv-FTLD. Moreover, the decreased CSF biomarkers^26,27^ and the basal ganglia and periventricular hypometabolism^28,29^ in the iNPH-bv-FTLD patients support a true underlying normal pressure hydrocephalus process. Furthermore, the difficulty in identifying Alzheimer’s disease in iNPH patients, notably due to misleading CSF biomarkers in iNPH (Aβ_42_, T-Tau and P-Tau are usually low due to abnormalities in protein drainage into the CSF),^27^ could have led us to underestimate the prevalence of iNPH among our Alzheimer’s disease patients. Lastly, our Memory Centre preferentially recruits patients with major cognitive and/or behavioural impairment. This could have contributed to reinforcing the prevalence of iNPH-bv-FTLD in our population, in comparison to the population usually seen in neurosurgical or neurological departments.

Further research is needed after this first observation. The increased prevalence of iNPH needs to be confirmed in other series of bv-FTLD patients. Neuropathological assessment of iNPH-bv-FTLD patients should provide interesting cues to elucidate the relationship between neurodegenerative processes and iNPH, especially since, beside the documented link with Alzheimer’s disease, a recent study suggests a high incidence of synucleinopathies among iNPH patients.^30^ Finally, more cases, a long-term follow-up, and standardized improvement criteria are mandatory to confirm the usefulness of a shunt in treating iNPH-bv-FTLD patients.

In conclusion, we report an unexpected high prevalence of iNPH cases among a bv-FTLD population, which raises the question of a possible pathophysiological link between these two entities initially thought to be unrelated. Clinicians are encouraged to look for iNPH in bv-FTLD patients, particularly in the case of associated gait or balance disturbances, as CSF shunting can be a useful treatment.

## Supplementary Material

fcac319_Supplementary_Data

## Data Availability

Data are available as Supplementary information.

## Abbreviations

Aβ_40_ =: 40 amino acid form of amyloid-β
Aβ_42_ =: 42 amino acid form of amyloid-β
ALS =: amyotrophic lateral sclerosis
bv-FTLD =: behavioural variant of frontotemporal lobar degeneration
CGI-C =: Clinical Global Impression of Change
DESH =: disproportionately enlarged subarachnoid space hydrocephalus
FDG-PET =: 18-F-fluorodeoxyglucose PET
iNPH =: idiopathic normal pressure hydrocephalus
HMPAO-SPECT =: hexa-methyl-propyl-amineoxime-single photon emission computed tomography
LP =: lumbar puncture
MMSE =: mini-mental state examination
MoCA =: Montreal Cognitive Assessment
MRRCN =: Memory Resource and Research Centre of Nantes
P-Tau =: phosphorylated Tau^181P^
SPECT =: single photon emission computed tomography
T-Tau =: total Tau
VAS =: ventriculoatrial shunt
VPS =: ventriculoperitoneal shunt
VOI =: volume of interest
