# Supplementary material for: Idiopathic normal pressure hydrocephalus and frontotemporal dementia: an unexpected association
Source: Brain Commun. 2022 Dec 14;4(6):fcac319. doi: 10.1093/braincomms/fcac319 (PMC9897186; doi:10.1093/braincomms/fcac319)
Supplement: fcac319_Supplementary_Data [file fcac319_Supplementary_Data.zip › Supplementary Material.pdf]

Supplemental Data

Supplementary file 1. Material and methods: cerebrospinal fluid analysis

Lumbar punctures were performed using a 25-gauge needle, and CSF samples were collected in a 5-mL polypropylene tube. Each CSF sample was transferred at 4°C to the corresponding local laboratory within 4 hours after collection and was then centrifuged at 2,100 g for 10 minutes at 4°C. The CSF was aliquoted in 2-mL polypropylene tubes and stored at −80°C to await further analysis.

Supplementary Table 1. Demographical, clinical, and biological features of the bv-FTLD and Alzheimer disease populations seen in the Memory Resource and Research Centre of Nantes in 2019

	**Bv-FTLD (*n* = 69)**	**AD (*n* = 178)**	***P***
Men	39 (56.5%)	62 (34.8%)	<0.001
Age at the first evaluation (years) (mean ± SD)	65.7±8.3	73.1±8.3	<0.0001
Age in 2019 (years) (mean ± SD)	68.8±8.2	76.5±8.2	<0.0001
Duration of symptoms at first visit (years) (mean ± SD)	3.1±2.8	2.2±2.0	0.02
Duration of symptoms in 2019 (years) (mean ± SD)	5.6±4.2	5.5 ±3.5	ns
MMSE at diagnosis (mean ± SD)	23.0±4.3	21.9±5.3	ns
Bv-FTLD			
Genetically confirmed	4 (5.8%) 3 *C9Orf72* expansion, 1 *GRN* mutation		
Probable	54 (78.3%)		
Possible	11 (15.9%)		
Amyotrophic lateral sclerosis	2 (2.9%)		
Dosage of CSF biomarkers	62 (84.9%)	84 (47.8%)	
AD diagnosis supported by CSF biomarkers or amyloid-PET imaging	0	84	

Bv-FTLD: behavioural variant of frontotemporal dementia, AD: Alzheimer's disease, MMSE: mini mental state evaluation
Statistical differences are assessed using Fisher's exact test, Student's test or Mann Whitney test.

Supplementary Table 2. Demographical and clinical features of patients in the iNPH-bv-FTLD, bv-FTLD, and iNPH groups

	**iNPH-bv-FLDT (*n* = 9)**	**Bv-FTLD (*n* = 9)**	**iNPH (*n* = 9)**
Men	3 (33.3%)	6 (55.5%)	7 (77.8%)
Age at the first evaluation (years) (Median, IQR25-75)	67 [64.5;74)	65 [62;69]	73 [65.5;74]
Genetically definite bv-FTLD<br>Probable bv-FTLD<br>Possible bv-FTLD	0<br>8 (88.9%)<br>1 (11.1%)	2 (22.2%)<br>7 (77.7%)<br>0	NA
Duration of symptoms at first evaluation (years) (Median, I25-75)	1 [1;3]	2 [1;5]	2 [0.5;3]
Duration of symptoms at last evaluation (years) (Median, I25-75)	5 [2;8.5]	5.5 [3.25;6.75]	4 [3;6.5]
ALS	1 (11.1%)	0	0
MMSE at diagnosis (Median; IQR25-75)	23 [16;27]	24 [20.5;27]	27 [26;28]
Number of Raskovsky's clinical criteria at the last visit (Median, IQR25-75)	**6 [5;6]**	**6 [5;6]**	1 [1;2]
Behavioural disinhibition	**9 (100%)**	**8 (88.9%)**	2 (22.2%)
Apathy	**9 (100%)**	**7 (77.7%)**	2 (22.2%)
Loss of empathy	**8 (88.9%)**	**9 (100%)**	1 (11.1%)
Perseverative behaviours	**6 (66.7%)**	**9 (100%)**	0
Hyperorality	**8 (88.9%)**	**9 (100%)**	1 (11.1%)
Physical neglect	**9 (100%)**	**7 (77.7%)**	0
Gait disturbance	**9 (100%)**	0	**9 (100%)**
Falls	**8 (88.9%)**	0	**6 (66.7%)**
Use of cane, rollator, or wheelchair	**4 (44.4%)**	0	**4 (44.4%)**
Sphincter disorders	**8 (88.9%)**	1 (10%)	**8 (88.9%)**
Improvement of gait after LPs, measured with the CGI-C (Median, range)	**2 [1-4] (*n* = 7)**		**2 [1-4] (*n* = 9)**
MMSE before LPs (Median, IQR25-75)<br>MMSE after LPs (Median, IQR25-75)	20 [17;26]<br>21 [17;26]		26 [24.5;27.5]<br>26.5 [18.75;27.25]
MoCA before LPs (Median, IQR25-75)<br>MoCA after LPs (Median, IQR25-75)	12.5 [10.75;18.5]<br>15 [9;22.5]		22.5 [15.5;25.25]<br>24 [14;25.75]
FAB before LPs (Median, IQR25-75)<br>FAB after LPs (Median, IQR25-75)	6 [5;12]<br>9 [6;16]		14 [10.5;15.75]<br>13.5 [10.5;17.25]
Improvement of gait after shunt, measured with the CGI-C (Median, range)	**2 [1-4] (*n* = 5)**		**3 [2-4] (*n* = 5)**

Bv-FTLD: behavioural variant of frontotemporal dementia, AD: Alzheimer's disease, iNPH: idiopathic normal pressure hydrocephalus, MMSE: mini-mental state evaluation, MoCA: Montreal Cognitive Assessment, FAB: Frontal Assessment Battery, CGI-C: Clinical Global Impression of Change, LPs: lumbar punctures. CGI-C scores range from 1 (very much improved) to 4 (no change).

Supplementary Table 3. Morphological imaging features of patients in the iNPH-bv-FTLD, bv-FTLD, and iNPH groups

	**iNPH-bv-FLDT (*n* = 9)**	**Bv-FTLD (*n* = 9)**	**iNPH (*n* = 9)**	***P***
MRI/CT-scan	9/0	9/0	6 / 3	
Fronto-parietal gradient	6 (66.7%)	3 (33.3%)	3 (33.3%)	
Frontal and/or temporal atrophy	7 (77.8%)	5 (55.5%)	4 (44.4%)	
Asymmetric atrophy	4 (44.4%)	2 (22.2%)	2 (22.2%)	
Evans index (median, IQR25-75)	**0.41 [0.35;0.41]**	0.31 [0.29;0.33]	**0.43 [0.40;0.45]**	
Tight superior sulci at the convexity	**8 (88.9%)**	1 (11.1%)	**7 (77.8%)**	
Callosal angle (median, IQR25-75)	**60 [46 ;72]**	111 [101.5;129.5]	**69 [57;82]**	
Cingular sulcus sign	**4 (44.4%)**	0	**5 (55.5%)**	
Convexity of the third ventricular walls	**4 (44.4%)**	0	**4 (44.4%)**	
Dilated Sylvian fissures	7 (77.8%)	3 (33.3%)	5 (55.6%)	
Leucopathy				
FAZEKAS 0	0	3	3	
FAZEKAS 1	4	4	2	0.13
FAZEKAS 2	1	1	3	
FAZEKAS 3	4	1	1	
Deep lacunar infarcts (≤ 3)	2 (22.2%)	1 (11.1%)	0	ns

Bv-FTLD: behavioural variant of frontotemporal dementia, AD: Alzheimer's disease, iNPH: idiopathic normal pressure hydrocephalus. Statistical differences are assessed using Kruskal-Wallis or Fisher's exact test.

Supplementary Table 4. FDG PET and SPECT visual analysis of patients in **the iNPH-bv-FTLD, bv-FTLD, and iNPH groups**

		**iNPH-bv-FLDT (*n* =8/9)**	**Bv-FTLD (*n* = 9/9)**	**iNPH (*n* = 4/9)**	***P* iNPH-bv-FLDT vs bv-FTLD**
**FDG PET** / **SPECT**		5/3	6/3	2/2	
Antero-posterior gradient		**5 (62.5%)**	**6 (66.7%)**	1 (25%)	ns
Asymmetrical perfusion or metabolism		2 (25%)	4 (44.4%)	3 (75%)	ns
Frontal hypometabolism	Absent (> 85%)	0	0	1 (25%)	
	Mild (75-80%)	3 (37.5%)	3 (33.3%)	1 (25%)	ns
	Moderate (50-75%)	3 (37.5%)	5 (55.5%)	2 (50%)	
	Severe (< 50%)	2 (25%)	1 (11.1%)	0	
Temporal hypometabolism	Absent (> 85%)	0	0	0	
	Mild (75-80%)	3 (37.5%)	3 (33.3%)	1 (25%)	ns
	Moderate (50-75%)	4 (50%)	5 (55.5%)	3 (75%)	
	Severe (< 50%)	1 (12.5%)	1 (11.1%)	0	
Parietal hypometabolism	Absent (> 85%)	1 (12.5%)	0	0	
	Mild (75-80%)	4 (50%)	6 (66.6%)	2 (50%)	ns
	Moderate (50-75%)	3 (37.5%)	3 (33.3%)	2 (50%)	
	Severe (< 50%)	0	0	0	
Basal ganglia hypometabolism		**8 (100%)**	5 (55.5%)	**3 (75%)**	**0.08**
Subcortical periventricular hypometabolism	Absent (>25%)	**0 (0%)**	2 (22.2%)	**0**	
	Moderate (10-25%)	**2 (25%)**	6 (66.6%)	**2 (50%)**	**<0.01**
	Severe (<10%)	**6 (75%)**	1 (11.1%)	**2 (50 %)**	

Bv-FTLD: behavioural variant of frontotemporal dementia, AD: Alzheimer's disease, iNPH: idiopathic normal pressure hydrocephalus. Statistical differences between iNPH-bv-FTLD and bv-FTLD groups are assessed using Kruskal-Wallis or Fisher's exact test.

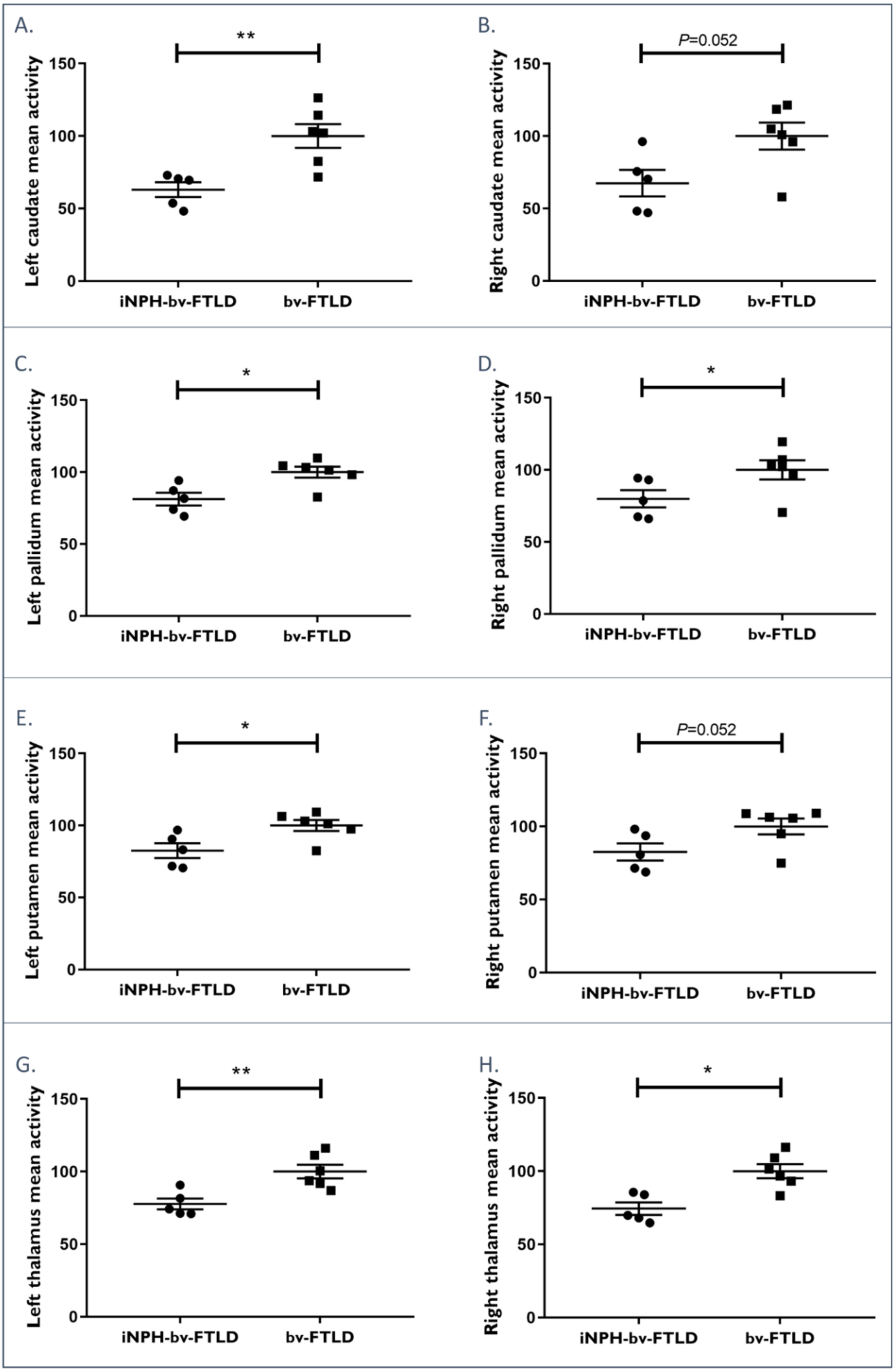

Supplementary Figure 1. Quantitative analysis of mean activity measured in basal ganglia VOI in patients who had an FDG-PET, from the iNPH-bv-FTLD and bv-FTLD groups. Statistical differences are assessed using Mann Whitney test; * P<0.05, ** P<0.01. Figure shows results as follows: left (A, U=1) and right (B, U=4) caudate, left (C, U=2) and right (D, U=3) pallidum, left (E, U=3) and right (F, U=4)) putamen, left (G, U=1) and right (H, U=2) thalami.

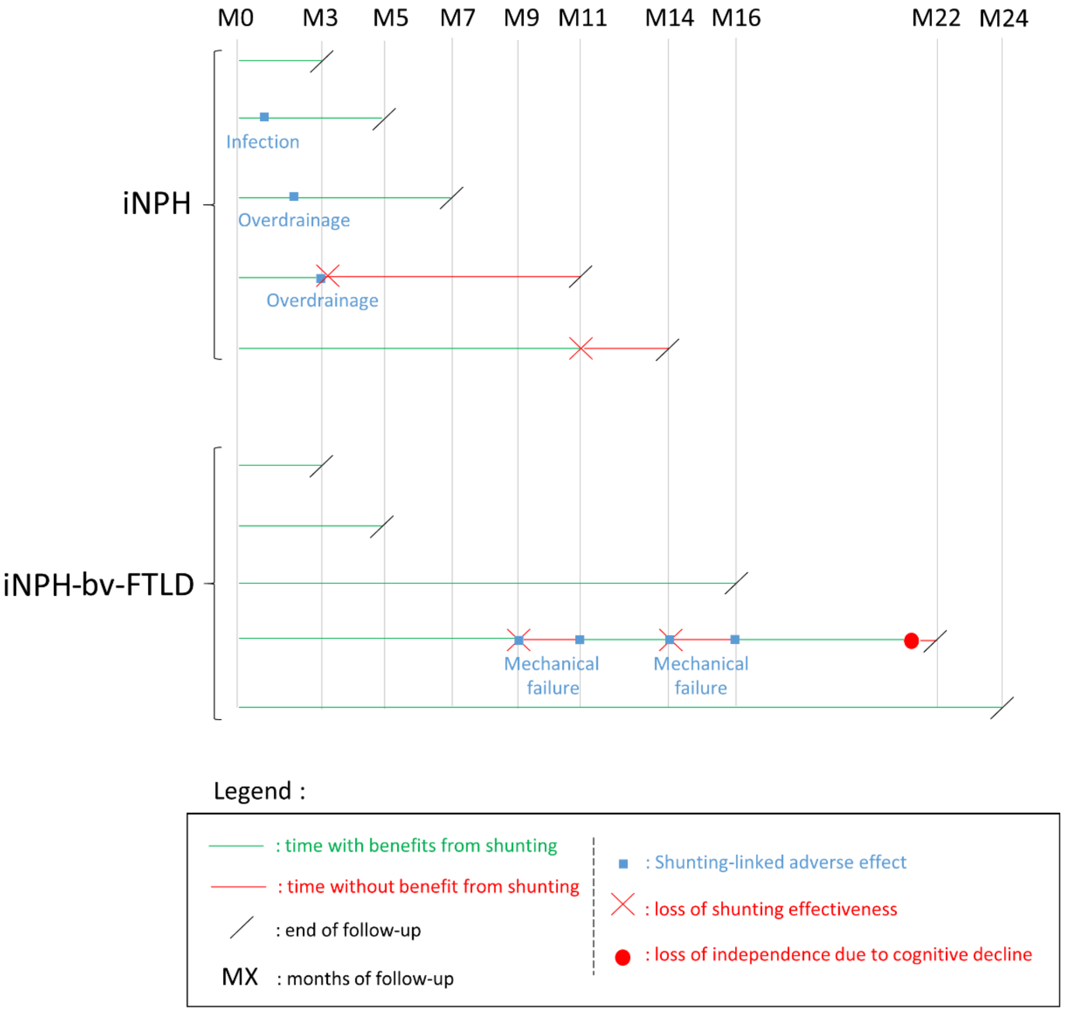

Supplementary Figure 2. Longitudinal follow-up of patients treated by ventriculoperitoneal (VPS) or atrial (VAS) shunt: median follow-ups are 7 and 16 months for iNPH and iNPH-bv-FTLD patients, respectively.
